# Cross-sectional and longitudinal analyses of urinary extracellular vesicle mRNA markers in urothelial bladder cancer patients

**DOI:** 10.1038/s41598-024-55251-x

**Published:** 2024-03-21

**Authors:** Taku Murakami, Keita Minami, Toru Harabayashi, Satoru Maruyama, Norikata Takada, Akira Kashiwagi, Haruka Miyata, Yasuyuki Sato, Ryuji Matsumoto, Hiroshi Kikuchi, Takashige Abe, Yoichi M. Ito, Sachiyo Murai, Nobuo Shinohara, Hiroshi Harada, Takahiro Osawa

**Affiliations:** 1 Research & Development, Showa Denko Materials (America), Inc., Irvine, CA USA; 2 Departments of Kidney Transplant Surgery and Urology, Sapporo City General Hospital, Sapporo, Japan; 3 Department of Urology, Hokkaido Cancer Center, Sapporo, Japan; 4 Department of Urology, Teine Keijinkai Hospital, Sapporo, Japan; 5 Department of Urology, Sapporo Keiyukai Hospital, Sapporo, Japan; 6 Department of Urology, Hokkaido University Hospital, N15 W7 Kita-ku, Sapporo, 060-8638 Japan; 7 Clinical Research and Medical Innovation Center, Hokkaido University Hospital, Sapporo, Japan

**Keywords:** Non-muscle invasive bladder cancer, Cancer recurrence, Biomarker, Extracellular vesicles, Exosome, mRNA, Cancer, Cancer screening, Tumour biomarkers, Urological cancer

## Abstract

We designed this multi-center prospective study with the following objectives: (1) the cross-sectional validation of extracellular vesicles (EV) mRNA markers to detect urothelial bladder cancer (UBC) before transurethral resection of bladder cancer (TURBT), and (2) the longitudinal validation of EV mRNA markers to monitor non-muscle invasive bladder cancer (NMIBC) recurrence after TURBT. EV mRNA markers evaluated in this study were *KRT17*, *GPRC5A*, and *SLC2A1* in addition to two additional markers from literatures, *MDK* and *CXCR2*, and measured by quantitative RT-PCR with normalization by a reference gene (*ALDOB*). Diagnostic performances of EV mRNA markers were compared to conventional markers. Regarding the first objective, we confirmed that EV mRNA biomarkers in urine were higher in UBC patients, particularly those with higher stage/grade tumors, than in those without UBC (n = 278 in total) and the diagnostic performance of EV mRNA *MDK* and *KRT17* outperformed conventional biomarkers with AUC 0.760 and 0.730, respectively. Concerning the second objective, we prospectively analyzed the time courses of EV mRNA markers while NMIBC patients (n = 189) (median follow-up 19 months). The expression of EV mRNA *KRT17* was significantly high in patients with recurrence, while it gradually decreased over time in those without recurrence (p < 0.01).

## Introduction

The National Cancer Institute Japan estimated about 23,000 new cases of bladder cancer in Japan in 2019^1^ and the American Cancer Society estimated about 82,000 cases of bladder cancer in 2023^2^. Bladder cancer is associated with subjective symptoms, including frequent urination and gross hematuria, and is frequently detected by imaging techniques, such as cystoscopy, ultrasonography, and CT. Between 70 and 80% of all bladder cancer patients are diagnosed with non-muscle invasive bladder cancer (NMIBC) and continue to receive surveillance following transurethral resection of bladder tumor (TURBT). Since the recurrence and progression rate of NMIBC is as high as 50 to 70%, those with bladder cancer history require lifelong monitoring of recurrence, which makes bladder cancer the most expensive cancer from diagnosis to treatment^3^. The gold standard surveillance method is cystoscopy, which is invasive and uncomfortable for patients. Urine cytology is a well-established non-invasive test due to its high specificity; however, its low sensitivity does not effectively exclude the presence of bladder cancer. Under these circumstances, there have been several reports of new non-invasive and sensitive tests using non-invasive biomarkers, but they are only used as an adjunctive diagnostic test to cystoscopy due to their low sensitivity and specificity^4,5^. Therefore, a combination of several methods might be a key to improve and personalize the surveillance strategy for patients with NMIBC.

Exosomes and microvesicles are membrane vesicles secreted at an elevated level in cancer patients^6^. These extracellular vesicles (EV) are known to be involved with intercellular communication through transporting biologically active molecules including mRNA, miRNA, DNA, and proteins between cells^7^. There are several studies using whole urine, urinary cells and exosomes for bladder cancer mRNA biomarkers^5,8^. One of the advantages to use EV mRNA as a diagnostic marker is that much knowledge has already been obtained regarding the expression patterns of mRNA in cancer tissues, urinary system organs, and immune system cells thanks to the recent multi-omics studies^9,10^. Therefore, when evaluating mRNA candidates in urinary EV as diagnostic markers, a selection process can be simple because there exists much data regarding putative biological functions, pathways, and expression patterns in various organs. In addition, assay development for mRNA is much easier than microRNA and protein due to the high sensitivity and specificity of real-time PCR method, which can greatly minimize a risk of non-specific amplification. In our previous cross-sectional study^11^, EV mRNA marker screening was conducted using urine from the patients with bladder cancer, and three EV mRNA markers, *SLC2A1*, *KRT17*, and *GPRC5A,* were identified and validated in a small single-center study. In particular, these three EV mRNA markers were 29.5, 20.6, and 18.2 times more highly expressed in urine from urothelial cancer patients than those in healthy controls, respectively.

In this prospective multi-center study, urinary EV mRNA markers were longitudinally evaluated before and after TURBT. Using the samples obtained before TURBT, we performed cross-sectional validation of the mRNA markers that we previously identified and analyzed^11^. Subsequently, the performance of these mRNA markers was validated as a predictor of tumor recurrence using longitudinal clinical data and urine samples. In addition, the performance of these EV mRNA-based urine markers was compared to cytology and other approved urinary markers including bladder tumor antigen (BTA), nuclear matrix protein 22 (NMP22) and UroVysion fluorescence in situ hybridization (FISH) assay^12^. We also developed the model for predicting tumor recurrence by combination of these several methods.

## Materials and methods

### Study design, clinical record and patient enrollment

**Figure 1:**
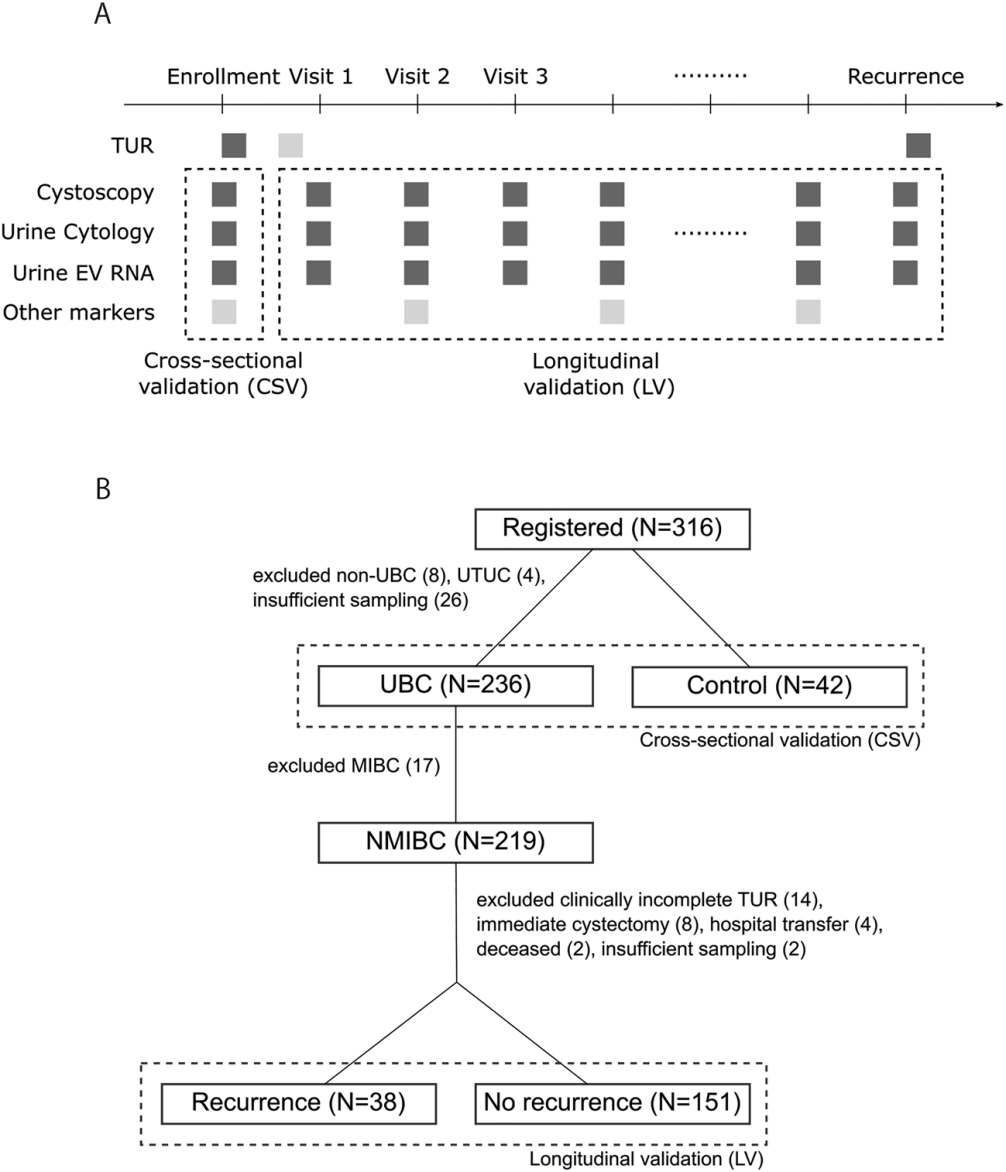
(**A**) Study design and sample collection schedule. Dark grey squares indicate mandatory procedures/sample collection, while light grey squares indicate optional procedures/sample collection. (**B**) Patient enrollment chart based on the study design. Numbers in parentheses indicate the numbers of corresponding patients/subjects. The two sample collection phases (CSV and LV) were indicated in perforated line boxes.

We designed this multicenter prospective study with the following two objectives: (1) cross-sectional validation (CSV) of the EV mRNA markers for urothelial bladder cancer (UBC) detection and (2) longitudinal validation (LV) of the EV mRNA markers for NMIBC recurrence monitoring (Fig. 1A).

To achieve these objectives, we registered patients at our institutes prior to their scheduled TUR surgeries and collected urine samples for marker validation (CSV phase) (Fig. 1A). Once pathologically confirmed as their tumors are NMIBC (i.e., LGTa, HGTa, T1, any Tis by the NCCN risk categories), we enrolled and followed up these patients for recurrence monitoring by the EV mRNA markers (LV phase) (Fig. 1A).

After the initial TURBT, indications for a second TURBT, intravesical therapy (Bacillus Calmette–Guérin (BCG) or intravesical chemotherapy (IVC)) after TURBT, and surveillance frequency until recurrence were based on each institution and urologist’s discretion according to the current clinical guideline^13^. Patients’ clinical data were prospectively recorded by urologists using a clinical record form including height, weight, gender, smoking status, previous UBC and other cancer history, clinical data (blood, urine, cystoscopy, urine cytology), pathology result, intravesical therapy information including a type of therapy (BCG and/or IVC), etc. The records were maintained and updated at every outpatient visit through the study and its integrity was reviewed by board certified urologists. Urine samples were collected during office visits for biomarker measurement and stored at −80 °C and shipped to our laboratory with dry ice unless otherwise noted.

Bladder tumor was diagnosed by cystoscopy, urine cytology and pathological diagnosis of resected tumors. Pathologic staging was reported according to the Union for International Cancer Control (UICC) staging system (7th edition)^14^. Tumor grade was determined by the World Health Organization 2004 criteria^15^. The patients were categorized into four risk groups using the NCCN guideline: LGTa, HGTa, T1, and any Tis^16^. Urine cytology specimens were evaluated by a cytopathologist in each institution following its standard operational procedure according to the Papanicolaou procedure^17^ or the Paris System^18^. Cytopathological findings were divided into negative cytology, suspicious cytology, or positive cytology as follows: (1) Negative cytology: Classes I and II in the Papanicolaou procedure or “Negative for high-grade urothelial carcinoma”, “Atypical urothelial cells” in the Paris System, (2) Suspicious cytology: Classes III and IV in the Papanicolaou procedure or “Suspicious for high-grade papillary urothelial carcinoma” in the Paris System, and (3) Positive cytology: Class V in the Papanicolaou procedure or “Low-grade urothelial neoplasm” and “High-grade urothelial carcinoma” in the Paris System. Microhematuria was defined as positive for greater than or equal to 5 red blood cells per high power field and negative for less than 5 cells.

We registered 316 patients including control (42 subjects) between October 2017 and November 2021 (Fig. 1B). Non-UBC (benign tumor) (N = 8) and upper tract urothelial carcinoma (UTUC) (N = 4) were excluded due to out of study scope, and 26 UBC patients were excluded due to insufficient urine sampling, which resulted in 236 UBC patients. Following three groups of subjects were enrolled as control: (1) healthy subjects who had no sign for bladder and other cancer through routine checkups (N = 11), (2) patients who had previous BC history but no recurrence at least for 17 months (median 4 years) (N = 17), and (3) patients who were suspected for UBC but later pathologically confirmed as benign tumors or no malignant disease (N = 14). In total, we enrolled 236 UBC patients and 42 control subjects and collected urine samples prior to TUR for the CSV phase. For the LV phase, after excluding 17 muscle invasive bladder cancer (MIBC) patients, 219 patients with NMIBC were followed up for cancer recurrence. In addition, 30 patients were excluded due to clinically incomplete TUR (N = 14), immediate cystectomy (N = 8), hospital transfer (N = 4), deceased (N = 2), and insufficient sampling (N = 2). As a result, we obtained clinical data and samples from 189 patients in total (38 cancer recurrence and 151 no recurrence cases) for the LV phase.

### Central Europe cohort

For additional CSV, an independent validation cohort of 30 UBC patients was recruited prospectively in Central Europe between June 2021 and November 2021 through a commercial biospecimen procurement service (Discovery Life Sciences, IRB approval no. DLS-BB044-V.1). Clinical data including pathology and urine cytology results and urine samples were obtained as described above.

### EV mRNA assay

EV mRNA assay was performed as described previously^11,19^. Briefly, after being thawed, urine was centrifuged at 800xg for 15 min to remove cells and large debris. Ten mL urine supernatant was used for EV isolation and mRNA extraction by an ExoComplete tube kit (Showa Denko Materials, Tokyo, Japan) following the manufacturer’s protocol. cDNA was synthesized by qScript XLT cDNA SuperMix (Quantabio, MA, USA) with the following reaction protocol: 5 min at room temperature, followed by 1 h at 42 °C and 5 min at 85 °C. Quantitative PCR (qPCR) was done by SsoAdvanced Universal SYBR Green Supermix (Bio-rad, CA, USA). Primer sequences used in this study is available in Supplementary Table 1. qPCR was performed in a ViiA 7 Real-Time PCR System (Thermo Fisher Scientific, CA, USA) with the following protocol: 95 °C 10 min, followed by 40 cycles of 95 °C 30 s and 65 °C for 1 min and a melting curve analysis. Threshold cycle values were obtained from the instrument software and normalized by that of reference gene (*ALDOB*) using the delta Ct method.

### FDA cleared biomarker assays

UroVysion assay (Abbott, IL, USA) was performed by a commercial clinical laboratory (SRL, Tokyo, Japan) following its sample collection protocol: isolating urine sediments and shipping to the laboratory at 4 °C. NMP22 ELISA kit (Abbott, IL, USA) and BTA stat kit (Polymedoco, NY, USA) were purchased and the measurement was conducted following the manufacturers’ protocols.

### Data analysis

Data analysis was conducted by R unless otherwise noted^20^. Kaplan–Meier survival curve analysis was conducted by R. Statistical significance was determined by a log-rank test. Censored patients were indicated by crosses in the curves. ROC curve analysis including area under the curve (AUC), sensitivity, specificity, positive predictive value (PPV) and negative predicative value (NPV) calculation was done by pROC^21^. Optimum threshold for each marker was obtained at the nearest point of the ROC curve to the top-left corner in the CSV phase and applied to the following analysis in the LV phase. EV mRNA expression and other markers were analyzed by Welch’s t-test. Time point comparisons for each group were analyzed with mixed model repeat measure model by JMP Pro 16 (SAS Institute Inc., NC, USA). p value of < 0.05 was considered statistically significant. Graphs were prepared by ggplot2^22^.

### Study review, approval and consent to participate

This study was reviewed and approved by institution review boards at Hokkaido University Hospital (IRB approval no. 017-0036), Sapporo City General Hospital (IRB approval no. R02-059–704), Hokkaido Cancer Center (IRB approval no. 017-0036), Teine Keijinkai Hospital (IRB approval no. 2-017135-01), and Sapporo Keiyukai Hospital (IRB approval no. H29-26). Informed consent was obtained from all individual participants included in the study. All methods were performed in accordance with the relevant guidelines and regulations.

## Results

### Patient enrollment and clinical outcomes

UBC patients (N = 236) enrolled in this study include LGTa (27%), HGTa (29%), T1 (22%), any Tis (14%), and MIBC (7%) by the NCCN risk categories and their clinical characteristics were summarized in Table 1.

**Table 1:** Patients’ characteristics.

			UBC	Control
Patient, n			236	42
Age, median year (IQR)			73 (66.5—79.5)	70 (63.3—76.8)
Sex, n (%)	Female		73 (31%)	15 (36%)
	Male		163 (69%)	27 (64%)
Smoking (%)	No		79 (33%)	6 (14%)
	Yes		129 (55%)	6 (14%)
	N/A		28 (12%)	30 (71%)
Prior UBC history (%)	No		168 (71%)	37 (88%)
	Yes		68 (29%)	5 (12%)
		1 time	11	3
		2 times	17	
		3 times	16	1
		> 3 times	17	1
		N/A	7	
Tumor size, n (%)	≤ 3 cm		159 (67%)	
	> 3 cm		58 (25%)	
	N/A		19 (8%)	
Tumor number, n (%)	1		105 (44%)	
	2 to 7		94 (40%)	
	≥ 8		20 (8%)	
	N/A		17 (7%)	
Pathological stage (%)	pTa		151 (64%)	
	pT1		61 (26%)	
	pTis		7 (3%)	
	pT2 or higher		17 (7%)	
	with pTis		28 (12%)	
Tumor grade (%)	LG		71 (30%)	
	HG		165 (70%)	
Second TUR (%)	No		170 (72%)	
	Yes		66 (28%)	
Intravesical therapy	No		116 (49%)	
	Yes		120 (51%)	
		BCG	59	
		IVC	41	
		BCG + IVC	16	
		N/A	4	
NCCN risk category (%)	LGTa		64 (27%)	
	HGTa		69 (29%)	
	T1		52 (22%)	
	any Tis		34 (14%)	
	MIBC		17 (7%)	

IQR, interquartile range; UBC, urothelial bladder cancer; TUR, transurethral resection; BCG, Bacillus Calmette–Guérin; IVC, Intravesical chemotherapy; LG, low grade; HG, high grade; MIBC, muscle-invasive bladder cancer; N/A, not available.

**Figure 2:**
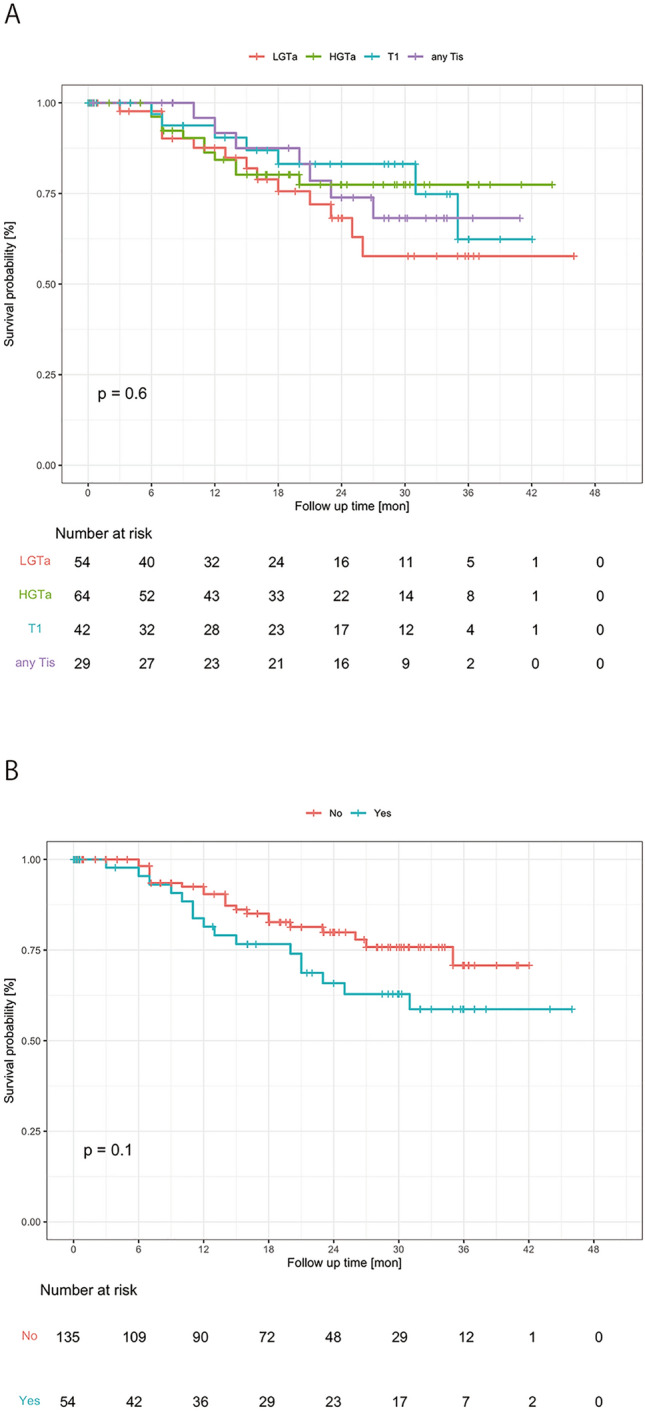
Recurrence-free survival curve analysis of the LV phase. A Kaplan–Meier survival curve analysis was conducted by NCCN risk categories (**A**) and bladder cancer history (**B**).

After our guideline-based surveillance during this study period (median follow up: 19 months), their 1-, 2- and 3-year recurrence-free survival were 87.7%, 75.4% and 66.9%, respectively (Table 2), and the NCCN risk category, UBC history, tumor size, tumor number, sex, and smoking status were not significant predictors for recurrence-free survival (Fig. 2A, B, Suppl Fig. 1A to 1D). The breakdown of initial TUR pathology and recurrent pathology are shown in Suppl Table 2.

**Table 2:** Follow-up results.

		Follow-up
Patients, n		189
Follow-up period, median months (IQR)		19 (9–30)
Recurrence-free survival, % (95% CI)	1 year	87.7% (82.6–93.2)
	2 years	75.4% (68.3–83.2)
	3 years	66.9% (57.6–77.8)
Recurrence, n (%)	No	151 (80%)
	Yes	38 (20%)
Recurrence pathology results (NCCN category)	LGTa	9 (24%)
	HGTa	10 (26%)
	T1	5 (13%)
	any Tis	7 (18%)
	MIBC	2 (5%)
	UTUC	2 (5%)
	Clinically diagnosed	3 (8%)

IQR, interquartile range; UBC, urothelial bladder cancer; CI, confidence interval; LG, low grade; HG, high grade; MIBC, muscle-invasive bladder cancer; UTUC, upper tract urothelial cancer.

### Cross-sectional marker validation

For the first study objective, we aimed for a CSV of the EV mRNA markers we identified in our previous study^11^. Although the previous study cohort was obtained in one of our institutes, the patients were recruited independently from the previous study.

First, we investigated if the previously identified reference gene, *ALDOB*, is useful to normalize the EV mRNA markers in urine in this study cohort. *ALDOB* and other candidate reference genes including *GAPDH* and *ACTB* were analyzed by ANOVA in the CSV phase (Suppl Table 2). *ALDOB* was not only one of the most highly expressed genes among the tested genes but also no change of its expression levels was observed among absence and presence of NMIBC/MIBC or among the NCCN risk categories, which satisfy the requirements of a reference gene. On the other hand, *GAPDH* and *ACTB*, were differentially expressed in urinary EV mRNA although their expression levels in urine was high, therefore those conventional reference genes were not ideal for normalization in this urinary EV mRNA analysis. These data corroborated our previous study result and re-confirmed that *ALDOB* expression is high and stable in urine EV independent of bladder tumor status and can be used to normalize the EV mRNA marker expression levels as a reference gene.

**Figure 3:**
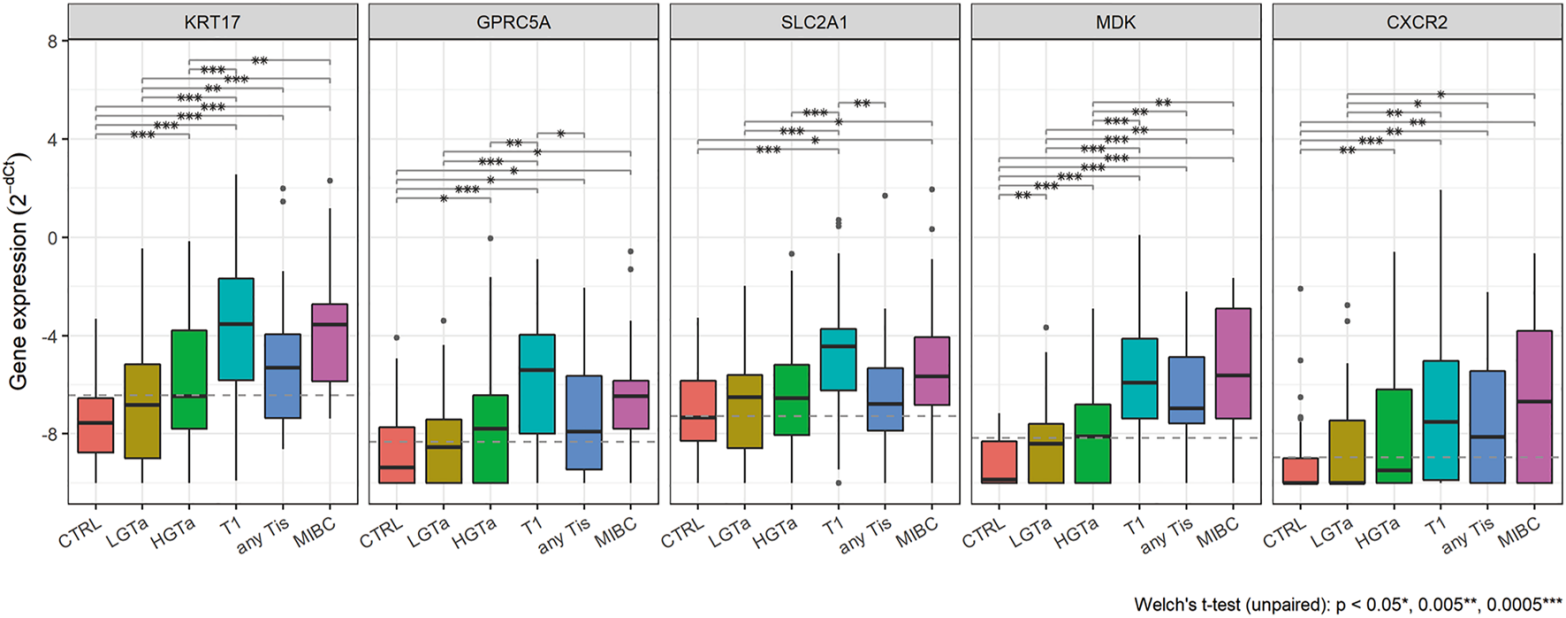
EV RNA expression in the CSV phase. Expression levels of EV RNA markers in the CSV phase are shown in boxplot graphs. Welch’s t-test was performed to compare the significance of differences between diagnostic categories based on NCCN risk categories: p < 0.05*, p < 0.005**, p < 0.0005***. Perforated lines indicate the best diagnostic threshold of each gene to detect UBC, which was defined in the top left corner of each ROC curve (Table 3).

Next, the EV mRNA marker expression, which was normalized by *ALDOB*, was analyzed in the CSV phase (Fig. 3). In this study, we focused on the three markers we identified in our previous study, *KRT17*, *GPRC5A* and *SLC2A1*^11^, in addition to the two additional markers from literatures, *MDK* and *CXCR2*^23^. These EV mRNA markers were highly expressed in UBC especially with higher stage/grade tumors such as T1, any Tis and MIBC compared to the control. In LGTa and HGTa, the EV mRNA expression especially *MDK* and *KRT17* was not as high as in T1, any Tis and MIBC however it was statistically significant compared to that in the control (Fig. 3). The NCCN risk categories do not include risk factors used in the AUA, EUA or EORTC risk categories, such as previous UBC history, tumor size and number. Therefore, the EV mRNA expression was further analyzed against these risk factors (Suppl Fig. 2). The EV mRNA expression was higher in those without previous UBC history, with larger tumors or with larger number of tumors than their counterparts. The result with UBC history was counterintuitive however this is because those without UBC history tend to have higher stage/grade tumors compared to those with UBC history who are under our surveillance for cancer recurrence. These data suggest that the EV mRNA expression level was associated with UBC stage, grade, size and number.

**Table 3:** Diagnostic performance in the CSV phase.

Markers	AUC (95% CI)	Sensitivity	Specificity	PPV	NPV
*MDK*	0.760 (0.699–0.821)	0.633	0.786	0.94	0.289
*KRT17*	0.730 (0.663–0.796)	0.62	0.81	0.945	0.288
Cytology	0.721 (0.614–0.829)	0.596	0.846	0.984	0.116
NMP22	0.692 (0.617–0.766)	0.56	0.824	0.945	0.257
*CXCR2*	0.674 (0.602–0.746)	0.534	0.81	0.937	0.248
Microhematuria	0.669 (0.562–0.777)	0.493	0.846	0.981	0.096
*GPRC5A*	0.660 (0.581–0.738)	0.606	0.69	0.912	0.25
BTA	0.659 (0.581–0.737)	0.628	0.69	0.912	0.266
*SLC2A1*	0.613 (0.531–0.695)	0.701	0.524	0.886	0.25

AUC, area under the curve; CI, confidence interval; PPV, positive predictive value; NPV, negative predictive value.

Diagnostic performance of these EV mRNA markers was estimated by ROC curve analysis and compared against that of urine cytology, microhematuria, and FDA cleared biomarkers (BTA and NMP22) (Table 3). Among the biomarkers, *MDK* showed the best AUC, 0.760, for the detection of UBC, followed by *KRT17* with AUC 0.730, and both EV mRNA markers outperformed conventional markers such as cytology (AUC 0.721), NMP22 (AUC 0.692), microhematuria (AUC 0.669) and BTA (AUC 0.659).

### Marker validation in the Central Europe cohort

To validate these EV mRNA markers further, we recruited a validation cohort of 30 subjects with prior UBC history in Central Europe. The patient characteristics of this cohort was summarized in Suppl Table 2. This cohort consists of 12 UBC subjects with LGHa (25%), HGTa (25%), T1 (42%) and MIBC (8%) as well as 18 control subjects who had previous UBC history and were at least six-month recurrence free. The EV mRNA markers as well as the other biomarkers were measured as described in the “Material and methods” section. In the Central Europe cohort, the EV mRNA markers were highly expressed in UBC patients compared to the control subjects (Suppl Fig. 3), which support the above CSV phase results (Fig. 3) as well as our previous study results^5^. In terms of marker performance, *MDK* and *KRT17* outperformed the other markers with AUC 0.824 and 0.736, respectively (Suppl Table 5), although statistical significance was not obtained in differential gene expression analysis among the NCCN risk categories (Suppl Fig. 3). Additionally, it is notable that BTA performed better in this cohort than in the CSV phase.

Taken together, these two CSV suggested that the urinary EV mRNA markers, especially *KRT17* and *MDK*, are promising biomarkers for the detection of UBC and may be superior to traditional biomarkers such as cytology, microhematuria and other FDA-cleared biomarkers although further validation is required.

### Longitudinal validation (LV) of the EV mRNA markers

**Figure 4:**
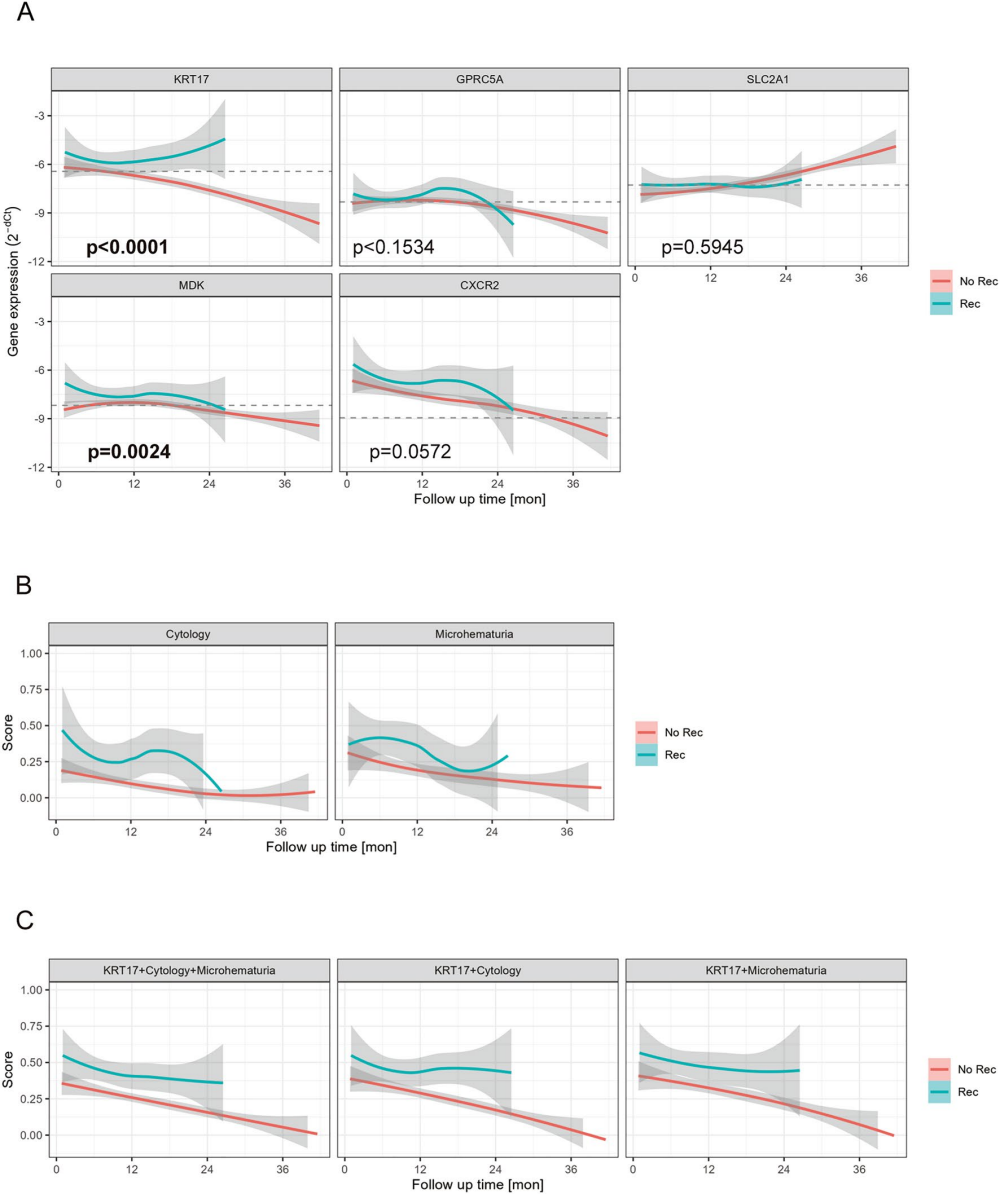
EV RNA expression and other markers in the LV phase. (**A**) Loess trendline curve analysis of EV RNA marker expression levels in the LV phase. Red lines indicate patients with no recurrence (N = 151) and blue lines indicate patients with cancer recurrence (N = 38). Follow-up times indicate months after first TUR. Perforated lines indicate the best threshold of each gene to detect UBC in the CSV phase (Table 3). (**B**) Loess trendline curve analysis of cytology and microhematuria. Score 1 was assigned for test positives and score 0 for test negatives. Definitions of test positives and negatives are described in the “Material and methods” section. (**C**) Loess trendline curve analysis of the average scores of EV *KRT17* and cytology/microhematuria. Regarding EV *KRT17*, score 1 was assigned for test positives when the expression level was above the threshold (perforated line in Fig. 4A) and score 0 for test negatives when the expression level was below the threshold. Average scores were obtained by arithmetic means of each score for EV *KRT17*, cytology, and/or microhematuria.

For the second study objective, we aimed for a LV of the EV mRNA markers in a surveillance setting after TURBT, assuming such markers will help earlier detection of cancer recurrence and/or reduce a frequency of cystoscopy procedures eventually. In this LV phase, urine samples were collected for the EV mRNA marker measurement (median 5 samples per patient) while the enrolled patients were under our guideline-based surveillance for cancer recurrence (Fig. 1A). Time courses of these marker expression in the LV phase were analyzed by Loess trend-line analysis (Fig. 4). For those without cancer recurrence during the study period (N = 151, “No Rec”), the expression of the *KRT17*, *GPRC5A*, and *MDK* remained similar to the thresholds for UBC detection, which was predetermined in the CSV phase of the study (Table 3), almost for 12 months after the TURBT, and gradually decreased over time and went below the thresholds only after 12–24 months. On the other hand, the time courses of *SLC2A1* and *CXCR2* indicated disappointing results indicating those markers may not be helpful to monitor the cancer recurrence. *SLC2A1* expression continuously increased over time. *CXCR2* expression was at least fourfold higher than the threshold for UBC detection immediately after the TURBT and decreased over time and required 30 months to go below the threshold.

To understand why these EV mRNA markers were expressed at the levels near the best thresholds for UBC detection for more than 12 months after TUR when tumors were supposedly eradicated, “No Rec” group was split into sub-groups by TUR pathology results, tumor size, tumor number or post-TUR therapies and time courses of the EV mRNA expression was compared among the sub-groups (Suppl Fig. 4). Although overall trends are similar to each other, several distinct patterns were observed. For example, *KRT17* and *CXCR2* expression levels were elevated in those who had more aggressive tumors during the first 12 months after TUR, i.e., LGTa < HGTa = any Tis < T1. Similarly, the EV mRNA expression was elevated in those with larger or greater number of tumors than those with smaller or less (Suppl Fig. 4B, 4C). These observations are interesting because the EV mRNA expression seems to be affected by tumor status even after tumors were eradicated, and more aggressive tumors leave the EV mRNA elevated even after TUR. Lastly, second TUR and intravesical therapy, which are generally provided during the first 6 months after initial TUR, seem to increase the EV mRNA expression levels higher may be due to more aggressive tumors or intravesical immune response, though it is inconclusive whether these therapies directly affect the expression levels (Suppl Fig. 4D).

On the other hand, for those with cancer recurrence in the LV phase (N = 38, “Rec”), *KRT17* expression was significantly elevated compared to not only the best threshold for UBC detection but also the expression level in the “No Rec” throughout the study period (p < 0.0001) (Fig. 4A). The difference of *KRT17* expression between “Rec” and “No Rec” increased over time especially after about 12 months. In addition, *MDK* was also expressed significantly higher in the “Rec” group than in the “No Rec”, time to time (p = 0.0224). For comparison, time courses of the cytology and microhematuria results in categorical scores (0 for negatives and 1 for positives) were analyzed (Fig. 4B). In the “No Rec” group, both cytology and microhematuria were close to score 0, or mostly negative, throughout the time, while BTA results were close to score 0.5, a mixture of positives and negatives, during the first 6 months after TUR and gradually decreased to score 0 over time. In the “Rec” group, on the other hand, cytology, microhematuria and BTA scores exceeded those in the “No Rec” group throughout the time.

Diagnostic performance of the EV mRNA markers for cancer recurrence detection was evaluated in the LV phase (Table 4A). *KRT17* outperformed the other markers with AUC 0.653 and NPV 91.6%. Compared to the cytology (AUC 0.603 and NPV 86.6%), *KRT17* could be a good alternative and may be useful to rule out those with no risk of cancer recurrence because of the relatively high NPV. Other EV mRNA such as *CXCR2* and *MDK* performed better than cystoscopy with AUC 0.628 and 0.606, respectively, while BTA and UroVysion were less promising with AUC 0.565 and 0.488, respectively. Since the EV mRNA expression remained close to the threshold levels for 12–24 months after the TUR, diagnostic performance was evaluated in the LV phase excluding the first 6 or 12 months after the TUR (Table 4B, C). Diagnostic performance of *KRT17* improved to AUC 0.667 and NPV 93.8% by excluding the first 6 months and AUC 0.700 and NPV 96.6% by excluding the first 12 months. Thus, *KRT17* could be used to rule out those without cancer recurrence more accurately especially after 6 to 12 months after TUR.

**Table 4:** Diagnostic performance in the LV phase.

A. Follow-up period (≥ 0 month)					
Markers	AUC (95% CI)	Sensitivity	Specificity	PPV	NPV
*KRT17*	0.653 (0.601–0.706)	0.764	0.519	0.243	0.916
*CXCR2*	0.628 (0.572–0.683)	0.664	0.594	0.248	0.898
NMP22	0.625 (0.300–0.950)	0.5	0.75	0.5	0.75
*MDK*	0.606 (0.548–0.664)	0.555	0.64	0.237	0.877
Cytology	0.603 (0.555–0.651)	0.283	0.924	0.426	0.866
Microhematuria	0.583 (0.534–0.633)	0.337	0.83	0.287	0.861
BTA	0.565 (0.473–0.658)	0.548	0.583	0.397	0.721
*GPRC5A*	0.561 (0.504–0.618)	0.582	0.558	0.21	0.869
*SLC2A1*	0.531 (0.476–0.586)	0.536	0.521	0.184	0.848
UroVysion	0.488 (0.464–0.512)	1	0	0.109	NaN
*KRT17* + cytology + microhematuria	0.697 (0.641–0.753)	0.8	0.536	0.25	0.933
*KRT17 *+ cytology	0.671 (0.614–0.727)	0.719	0.59	0.257	0.914
*KRT17* + microhematuria	0.655 (0.600–0.710)	0.723	0.565	0.249	0.911

B. Follow-up period (≥ 6 months)					
Markers	AUC (95% CI)	Sensitivity	Specificity	PPV	NPV
*KRT17*	0.667 (0.607–0.726)	0.79	0.548	0.233	0.938
*CXCR2*	0.620 (0.555–0.685)	0.642	0.621	0.227	0.909
Cytology	0.600 (0.547–0.653)	0.264	0.936	0.422	0.878
*MDK*	0.595 (0.530–0.661)	0.531	0.642	0.205	0.888
Microhematuria	0.576 (0.520–0.633)	0.315	0.838	0.25	0.877
BTA	0.569 (0.448–0.690)	0.458	0.68	0.407	0.723
*GPRC5A*	0.565 (0.500–0.630)	0.568	0.58	0.19	0.886
*SLC2A1*	0.547 (0.483–0.612)	0.469	0.625	0.178	0.872
UroVysion	0.488 (0.464–0.512)	1	0	0.109	NaN
*KRT17 *+ cytology + microhematuria	0.697 (0.634–0.760)	0.788	0.562	0.233	0.94
*KRT17* + cytology	0.675 (0.612–0.738)	0.7	0.618	0.243	0.922
*KRT17* + microhematuria	0.660 (0.598–0.722)	0.718	0.584	0.228	0.924

C. Follow-up period (≥ 12 months)					
Markers	AUC (95% CI)	Sensitivity	Specificity	PPV	NPV
*KRT17*	0.700 (0.620–0.779)	0.825	0.573	0.182	0.966
*CXCR2*	0.628 (0.534–0.722)	0.575	0.712	0.187	0.936
Cytology	0.625 (0.549–0.700)	0.297	0.952	0.423	0.92
*MDK*	0.618 (0.527–0.709)	0.625	0.651	0.171	0.938
*GPRC5A*	0.615 (0.524–0.706)	0.625	0.597	0.152	0.932
BTA	0.611 (0.413–0.810)	0.444	0.778	0.5	0.737
*SLC2A1*	0.603 (0.516–0.690)	0.5	0.674	0.15	0.921
Microhematuria	0.530 (0.463–0.596)	0.189	0.87	0.143	0.904
UroVysion	0.486 (0.458–0.514)	1	0	0.103	NaN
*KRT17* + cytology + microhematuria	0.718 (0.637–0.799)	0.8	0.603	0.19	0.963
*KRT17* + cytology	0.714 (0.629–0.800)	0.722	0.659	0.198	0.953
*KRT17* + microhematuria	0.667 (0.586–0.748)	0.722	0.625	0.179	0.952

AUC, area under the curve; CI, confidence interval; PPV, positive predictive value; NPV, negative predictive value.

To further improve the diagnostic performance, we employed a simple algorithmic analysis of *KRT17* in combination with cytology and/or microhematuria results. For *KRT17*, we assigned score 1 (positive) when its expression level is above the threshold determined in the CSV phase and score 0 (negative) when the expression is below the threshold. Averaged scores of *KRT17*, cytology and/or microhematuria were analyzed, which represents the average number of positive markers out of each combination (Fig. 4C). Diagnostic performance was improved to AUC 0.697, 0.697 and 0.718 by adding cytology and hematuria results to *KRT17* although NPV wasn’t improved (Table 4).

**Figure 5:**
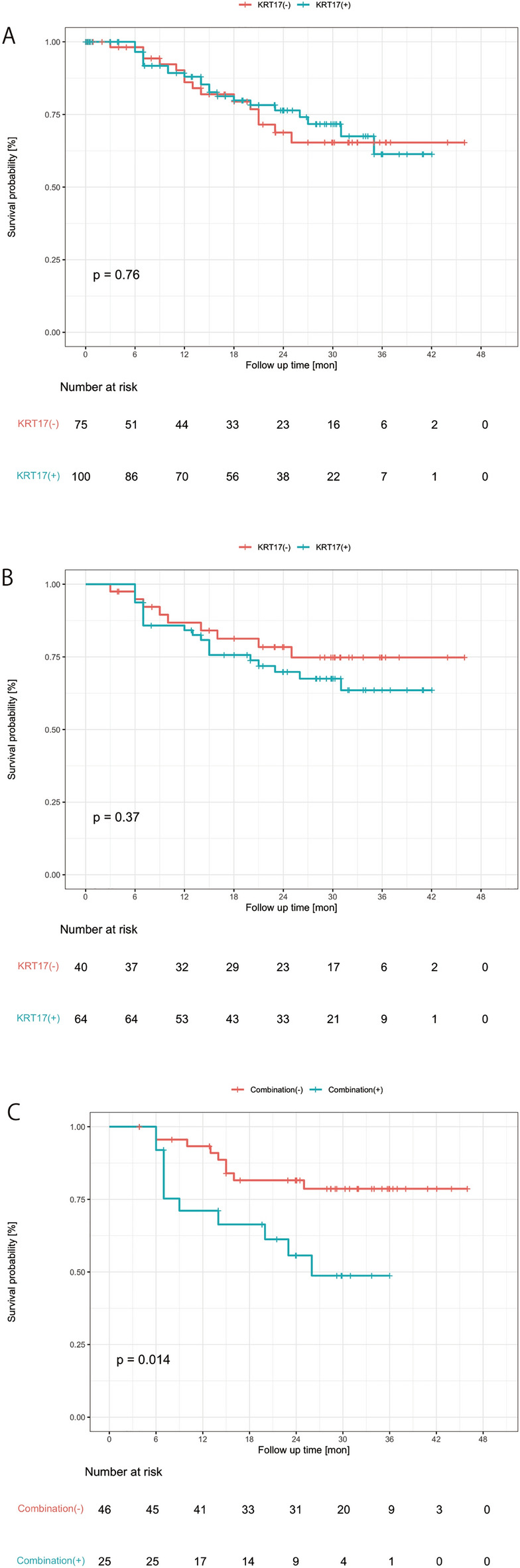
Recurrence-free survival curve analysis of the LV phase. Patients were stratified by EV *KRT17* in urine obtained before TUR (CSV phase) (**A**) or during the first 6 months after TUR (LV phase) (**B**) using the threshold defined in the CSV phase (Table 3), and recurrence-free survival was compared between EV *KRT17* positives and negatives. Patients were also stratified by the average score of EV *KRT17*, cytology, and microhematuria during the first 6 months after TUR (LV phase) using a cut-off of 0.5 (**C**). The significance of differences was assessed by the Log-rank test. Censored patients were indicated by crosses in the curves. (**A**) EV *KRT17*, CSV phase (**B**) EV *KRT17*, LV phase (<6 months) (**C**) Combination (EV *KRT17*+Cytology+Microhematurial), LV phase (<6 months)

Lastly, predictive value of *KRT17* was investigated. First, *KRT17* in urine obtained prior to TUR (CSV phase) was not predictive for the post-TUR recurrence-free survival (Fig. 5A). *KRT17* during the first 6 months after TUR (LV phase) was not predictive either (log rank p > 0.05), however at least those with *KRT17* positives indicated slightly poor prognosis compared to those with *KRT17* negatives (Fig. 5B). On the other hand, the average score of *KRT17*, cytology and microhematuria during the first 6 months after TUR was clearly able to predict recurrence-free survival (Fig. 5C) while each marker alone did not (Fig. 5B, Suppl Fig. 5A, B). Therefore, *KRT17* in combination with other conventional biomarkers may have a predictive value of cancer recurrence although further validations are necessary.

## Discussion

We designed this study with the two objectives: 1. CSV of the EV mRNA markers for UBC detection and 2. LV of the EV mRNA markers for NMIBC recurrence monitoring. For the first objective, we were able to confirm the EV mRNA markers were highly expressed in urine from UBC patients especially with higher stage/grade tumors compared to those without UBC in the CSV phase (N = 278 in total) and Central Europe cohorts (N = 30 in total) (Fig. 3, Suppl Fig. 1). We also confirmed that the EV mRNA markers especially *MDK* and *KRT17* outperformed urine cytology and other conventional markers in both cohorts (Table 3, Suppl Table 4). Those data support the validity of the EV mRNA markers for UBC detection. For the second objective, we investigated the time courses of the EV mRNA markers prospectively while NMIBC patients (N = 189) were under our surveillance in the LV phase. One of the EV mRNA markers, *KRT17*, was confirmed to be expressed higher in those with cancer recurrence after TURBT until recurrence, while its expression gradually decreased over a time for those without cancer recurrence during the study period (Fig. 4A). Indeed, NPV of *KRT17* obtained in this study was 91.6%, 93.8% and 96.6% for > 0 month, > 6 months and > 12 months after TURBT, respectively, thus it may be helpful to reduce surveillance frequency/duration using this marker although further validation studies are necessary (Table 4). These data suggest that the EV mRNA markers especially, *KRT17*, is a promising biomarker for UBC detection and recurrence monitoring.

It is intriguing that *KRT17*, and other mRNA expression remained high for more than 12 months after TURBT in those with no recurrence despite tumors were eradicated by TURBT (Fig. 4A), which may hamper the use of these markers immediately after TURBT. Our analysis indicated that those who had more aggressive tumors or those who received second TURBT tend to have high EV mRNA expression even after TURBT (Suppl Fig. 4). In those who had cancer recurrence, *KRT17* expression was high already before cancer recurrence was detected by cystoscopy. Thus, the source of *KRT17* and other markers to be further investigated in the future study. On the other hand, these data suggest *KRT17* may be clinically more useful for selected patient populations such as those with less aggressive tumors.

*KRT17* has been reported to be overexpressed in many types of cancer including UBC, breast cancer, colon cancer, non-small lung cancer, cervical cancer, oral cancer, esophagus cancer, pancreatic cancer, etc. *KRT17* overexpression is associated with poor prognosis and cancer progression in non-small lung cancer^24^, colon cancer^25^ and other types of cancer^26^. To the contrary, Wu et al. reported that *KRT17* low expression in UBC is associated with poor overall and progression-free survivals by histochemistry analysis. Also, *KRT17* is expressed higher in less aggressive tumors than more aggressive ones^27^. In this study, EV *KRT17* and cytology/microhematuria scores during the first 6 months after TURBT (LV phase) were able to predictive recurrence-free survival (Fig. 5C) and at least those with high EV *KRT17* expression indicated a relatively poor survival though statistical significance wasn’t obtained (Fig. 5B). The discrepancy between the study by Wu et al.^27^ and this study may be due to the difference between tumor *KRT17* and urinary EV *KRT17*. Babu et al.^28^ recently reported that KRT17 protein is over expressed in urinary cells and tumors in bladder cancer patients and is a highly accurate biomarker for bladder cancer. These accumulating data support the validity of *KRT17* as a marker for bladder cancer.

Achieving the good performance for predicting recurrence is challenging, because the recurrent lesions under the follow-ups are usually very small to discharge EVs into urine. This is consistent with the result in which most of tumor size was less than 3 cm at the time of the recurrence. However, *KRT17* can discriminate the tumor recurrence after 12 months after surgery. The discrimination ability was improved as the follow-up was prolonged. This discrimination ability might be due to the elimination of insignificant perioperative effect. Additionally, we created a simple algorithm combining *KRT17* and conventional biomarkers such as urine cytology and microhematuria. We chose these two markers because of the high sensitivity of microscopic hematuria and the high specificity of urine cytology. In fact, this combination improved the diagnostic performance. Furthermore, with the recent advancement in this field, recurrence monitoring could be further improved in combination with other biomarkers such as urinary cells and circulating tumor DNA. Urinary cells could be analyzed from whole urine together with EV RNA, therefore new urinary cell RNA assay^23^ and KRT17 immunohistochemistry^28^ could be complementary to EV RNA assay though further clinical validation is necessary.

There are several limitations in this study. First, despite overall trend of the EV mRNA expression time course looks promising, the time course in each case looks noisy and difficult to take actual clinical actions based on the biomarker data yet. In the future, larger scale and long-term validation studies are necessary. In addition, new laboratory technologies, such as droplet digital PCR, which allow more accurate quantification, especially at very low concentrations, may overcome this problem, as mRNA expression levels in urinary EV are much lower than in tissue. In addition, the predictors with significant differences in the other reports were not necessarily significant factors in our study because a portion of patients were excluded due to radical cystectomy. Diagnostic performance was compared to UroVysion and NMP22, however sample sizes of these assays especially in the LV phase were limited (N = 46 and N = 12, respectively). EV *ALDOB* was selected for a reference gene to normalize EV mRNA marker expression levels because we confirmed EV *ALDOB* level was stable in our studies. However, *ALDOB* is highly expressed in kidney therefore EV *ALDOB* levels in urine may fluctuate depending on the patients’ kidney functions, which we were not able to investigate in this study because of limited clinical data.

Taken together, these data suggest that the EV mRNA markers especially, *KRT17*, is a promising biomarker for UBC detection and recurrence monitoring though further validations especially in real-life clinical settings are necessary.

### Supplementary Information

Supplementary Table 1.

Supplementary Table 2.

Supplementary Table 3.

Supplementary Table 4.

Supplementary Table 5.

Supplementary Figure 1.

Supplementary Figure 2.

Supplementary Figure 3.

Supplementary Figure 4.

Supplementary Figure 5.

## Data Availability

All data generated or analysed during this study are included in this published article and its supplementary information files.

## Abbreviations

EV: Extracellular vesicles
TUR: Transurethral resection of bladder tumor
AUC: Area under the curve
UBC: Urothelial bladder cancer
qPCR: Quantitative polymerase chain reaction
