# Supplementary material for: Cross-sectional and longitudinal analyses of urinary extracellular vesicle mRNA markers in urothelial bladder cancer patients
Source: Sci Rep. 2024 Mar 21;14:6801. doi: 10.1038/s41598-024-55251-x (PMC10957914; doi:10.1038/s41598-024-55251-x)
Supplement: Supplementary file 1 — Supplementary Table 1. [file 41598_2024_55251_MOESM1_ESM.docx]

**Supplementary Table 1. Primer sequences**

| Gene | Sense (5' to 3') | Antisense (5' to 3') |
| --- | --- | --- |
| ACTB | tttttcctggcacccagcacaat | tttttgccgatccacacggagtact |
| ALDOB | aaccaccattcaagggcttg | ttggcgttttcctggatagc |
| CXCR2 | aaactggcggatgctgttac | aacagcatgatcagcagtgg |
| GAPDH | cccactcctccacctttgac | cataccaggaaatgagcttgacaa |
| GPRC5A | gctcatgcttcctgactttgac | ttgtgagcagccaaaactcg |
| KRT17 | tggacaatgccaacatcctg | tcaaacttggtgcggaagtc |
| MDK | tacaatgctcagtgccaggag | tggcctttgctttggtcttg |
| SLC2A1 | cttcatcatcggtgtgtactgc | gaacacctgggcgatgagg |

Sequences of the PCR primer pairs used in this study were shown. Primer synthesis was conducted by Integrated DNA technologies (IA, USA).
