# Supplementary material for: Cross-sectional and longitudinal analyses of urinary extracellular vesicle mRNA markers in urothelial bladder cancer patients
Source: Sci Rep. 2024 Mar 21;14:6801. doi: 10.1038/s41598-024-55251-x (PMC10957914; doi:10.1038/s41598-024-55251-x)
Supplement: Supplementary file 2 — Supplementary Table 2. [file 41598_2024_55251_MOESM2_ESM.docx]

**Supplementary Table 2. Comparison of TUR and recurrence pathology**

|  | TUR pathology | | | |
| --- | --- | --- | --- | --- |
| Recurrence pathology | LGTa  (N=13) | HGTa (N=11) | T1  (N=7) | any Tis  (N=7) |
| LGTa | 5 (38%) | 1 (9%) | 2 (29%) | 1 (14%) |
| HGTa | 6 (46%) | 4 (36%) |  |  |
| T1 | 1 (8%) |  | 2 (29%) | 2 (29%) |
| any Tis | 1 (8%) | 1 (9%) | 2 (29%) | 3 (43%) |
| MIBC |  | 1 (9%) |  | 1 (14%) |
| UTUC |  | 2 (18%) |  |  |
| Clinically diagnosed |  | 2 (18%) | 1 (14%) |  |

For the patients who had cancer recurrence during the study period (N=38), TUR (Table 1) and recurrence (Table 2) pathologies were compared. The numbers indicate the patient number and those in parentheses do percentages for each NCCN risk category.
