# Supplementary material for: Cross-sectional and longitudinal analyses of urinary extracellular vesicle mRNA markers in urothelial bladder cancer patients
Source: Sci Rep. 2024 Mar 21;14:6801. doi: 10.1038/s41598-024-55251-x (PMC10957914; doi:10.1038/s41598-024-55251-x)
Supplement: Supplementary file 3 — Supplementary Table 3. [file 41598_2024_55251_MOESM3_ESM.docx]

**Supplementary Table 3. Analysis of variance for reference gene selection**

|  | Threshold cycle | | ANOVA, p value | |
| --- | --- | --- | --- | --- |
| Gene | mean | median | Absence/Presence of NMIBC/MIBC | NCCN risk category |
| ALDOB | 25.5 | 25.4 | n.s. | n.s. |
| GAPDH | 26.7 | 26.9 | 1.3 x 10^-10^ | 8.8 x 10^-7^ |
| ACTB | 27.0 | 27.1 | 5.4 x 10^-12^ | 2.7 x 10^-8^ |

ANOVA analysis of the EV RNA expression levels was conducted to select a reference gene for the EV RNA markers. Raw threshold cycle values were used for analysis. n.s.: not significant or p value > 0.05.
