# Supplementary material for: Cross-sectional and longitudinal analyses of urinary extracellular vesicle mRNA markers in urothelial bladder cancer patients
Source: Sci Rep. 2024 Mar 21;14:6801. doi: 10.1038/s41598-024-55251-x (PMC10957914; doi:10.1038/s41598-024-55251-x)
Supplement: Supplementary file 4 — Supplementary Table 4. [file 41598_2024_55251_MOESM4_ESM.docx]

**Supplementary Table 4. Patient characteristics of the Central Europe cohort**

|  |  | UBC | Control |
| --- | --- | --- | --- |
| Patient, n |  | 12 | 18 |
| Age, median year (IQR) |  | 67.5 (62.4 - 72.6) | 64 (56.4 - 71.6) |
| Sex, n (%) | Female | 1 (8%) | 4 (22%) |
|  | Male | 11 (92%) | 14 (78%) |
| Smoking (%) | No | 12 (100%) | 18 (100%) |
|  | Yes | 0 (0%) | 0 (0%) |
| Prior BC history (%) | No | 0 (0%) | 0 (0%) |
|  | Yes | 12 (100%) | 18 (100%) |
| Tumor size, n (%) | ≤ 3 cm | 9 (75%) |  |
|  | > 3 cm | 3 (25%) |  |
| Pathological stage (%) | pTa | 6 (50%) |  |
|  | pT1 | 5 (42%) |  |
|  | pT2 or higher | 1 (8%) |  |
| Tumor grade (%) | LG | 4 (33%) |  |
|  | HG | 8 (67%) |  |
| Second TUR (%) | No | 12 (100%) | 18 (100%) |
|  | Yes | 0 (0%) | 0 (0%) |
| NCCN risk category (%) | LGTa | 3 (25%) |  |
|  | HGTa | 3 (25%) |  |
|  | T1 | 5 (42%) |  |
|  | MIBC | 1 (8%) |  |

Patients’ characteristics enrolled in the Central Europe cohort was summarized in the table. IQR: interquartile range, UBC: urothelial bladder cancer, BC: bladder cancer, TUR: transurethral resection, BCG: Bacillus Calmette–Guérin, IVC: Intravesical chemotherapy, LG: low grade, HG: high grade, MIBC: muscle-invasive bladder cancer, N/A: not available.
