# Supplementary material for: Cross-sectional and longitudinal analyses of urinary extracellular vesicle mRNA markers in urothelial bladder cancer patients
Source: Sci Rep. 2024 Mar 21;14:6801. doi: 10.1038/s41598-024-55251-x (PMC10957914; doi:10.1038/s41598-024-55251-x)
Supplement: Supplementary file 5 — Supplementary Table 5. [file 41598_2024_55251_MOESM5_ESM.docx]

**Supplementary Table 5. Diagnostic performance in the Central Europe cohort**

| Markers | AUC (95% CI) | Sensitivity | Specificity | PPV | NPV |
| --- | --- | --- | --- | --- | --- |
| MDK | 0.824 (0.650-0.998) | 0.75 | 0.944 | 0.9 | 0.85 |
| BTA | 0.750 (0.593-0.907) | 0.833 | 0.667 | 0.625 | 0.857 |
| KRT17 | 0.736 (0.534-0.939) | 0.667 | 0.778 | 0.667 | 0.778 |
| SLC2A1 | 0.736 (0.550-0.922) | 0.667 | 0.722 | 0.615 | 0.765 |
| GPRC5A | 0.729 (0.539-0.920) | 0.75 | 0.722 | 0.643 | 0.812 |
| CXCR2 | 0.690 (0.493-0.886) | 0.583 | 0.833 | 0.7 | 0.75 |
| Cytology | 0.591 (0.471-0.710) | 0.182 | 1 | 1 | 0.654 |

Diagnostic performance of the markers studied in the Central Europe cohort was summarized in the table. AUC: area under the curve, CI: confidence interval, PPV: positive predictive value, NPV: negative predictive value.
