# Supplementary figures and images for: Cross-sectional and longitudinal analyses of urinary extracellular vesicle mRNA markers in urothelial bladder cancer patients
Source: Sci Rep. 2024 Mar 21;14:6801. doi: 10.1038/s41598-024-55251-x (PMC10957914; doi:10.1038/s41598-024-55251-x)

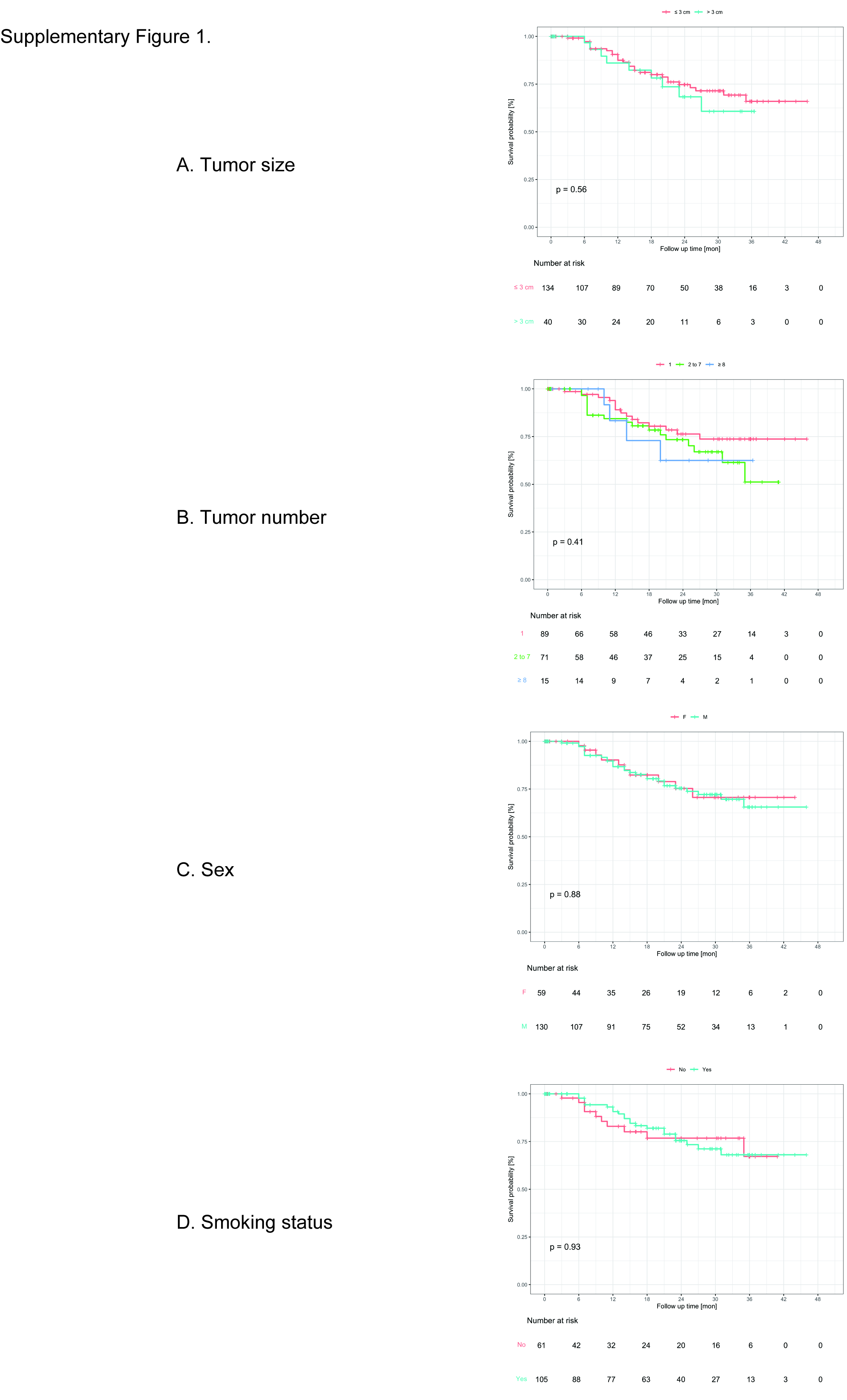

Supplement: Supplementary file 6 — Supplementary Figure 1. [file 41598_2024_55251_MOESM6_ESM.tif]

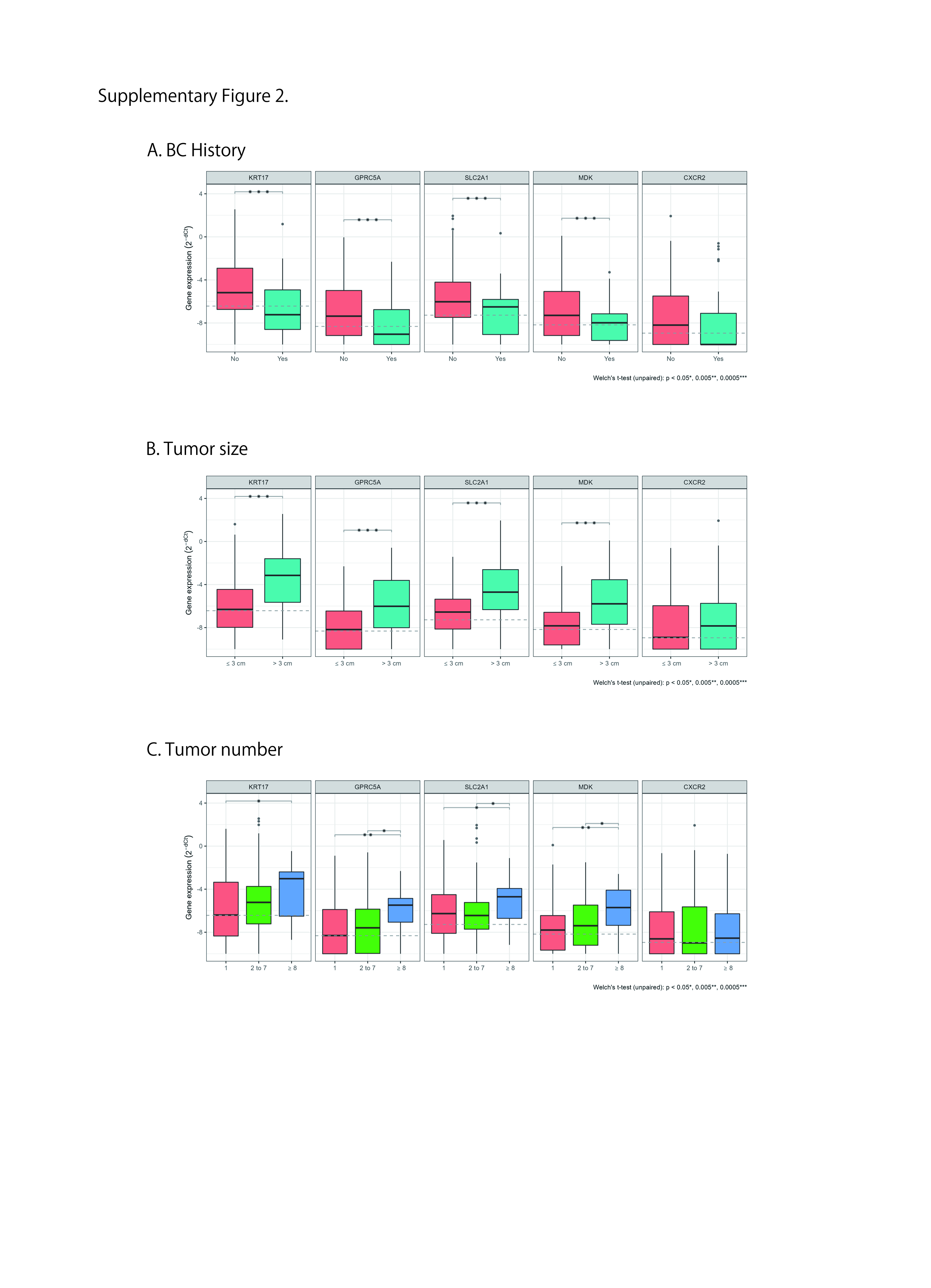

Supplement: Supplementary file 7 — Supplementary Figure 2. [file 41598_2024_55251_MOESM7_ESM.tif]

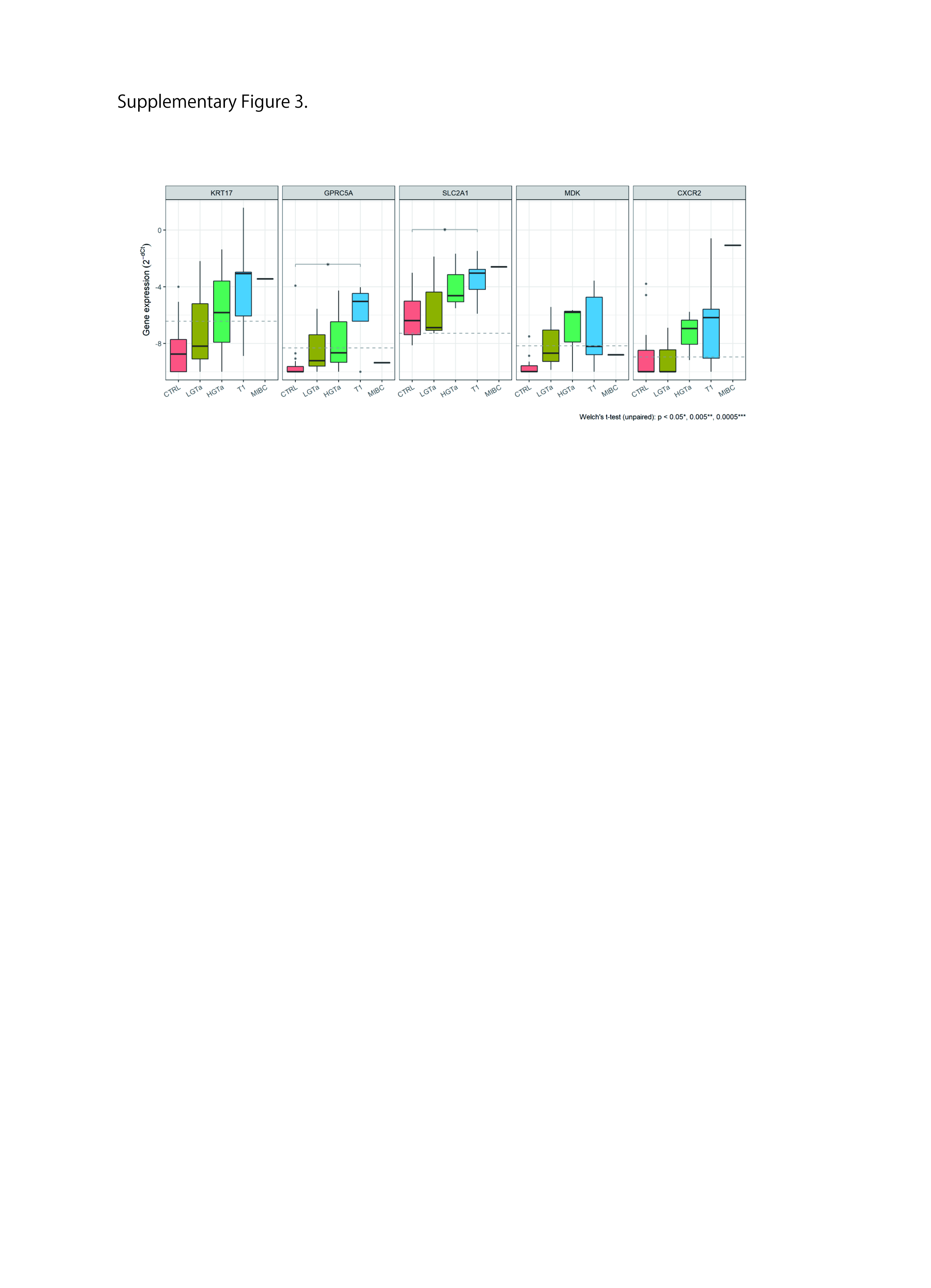

Supplement: Supplementary file 8 — Supplementary Figure 3. [file 41598_2024_55251_MOESM8_ESM.tif]

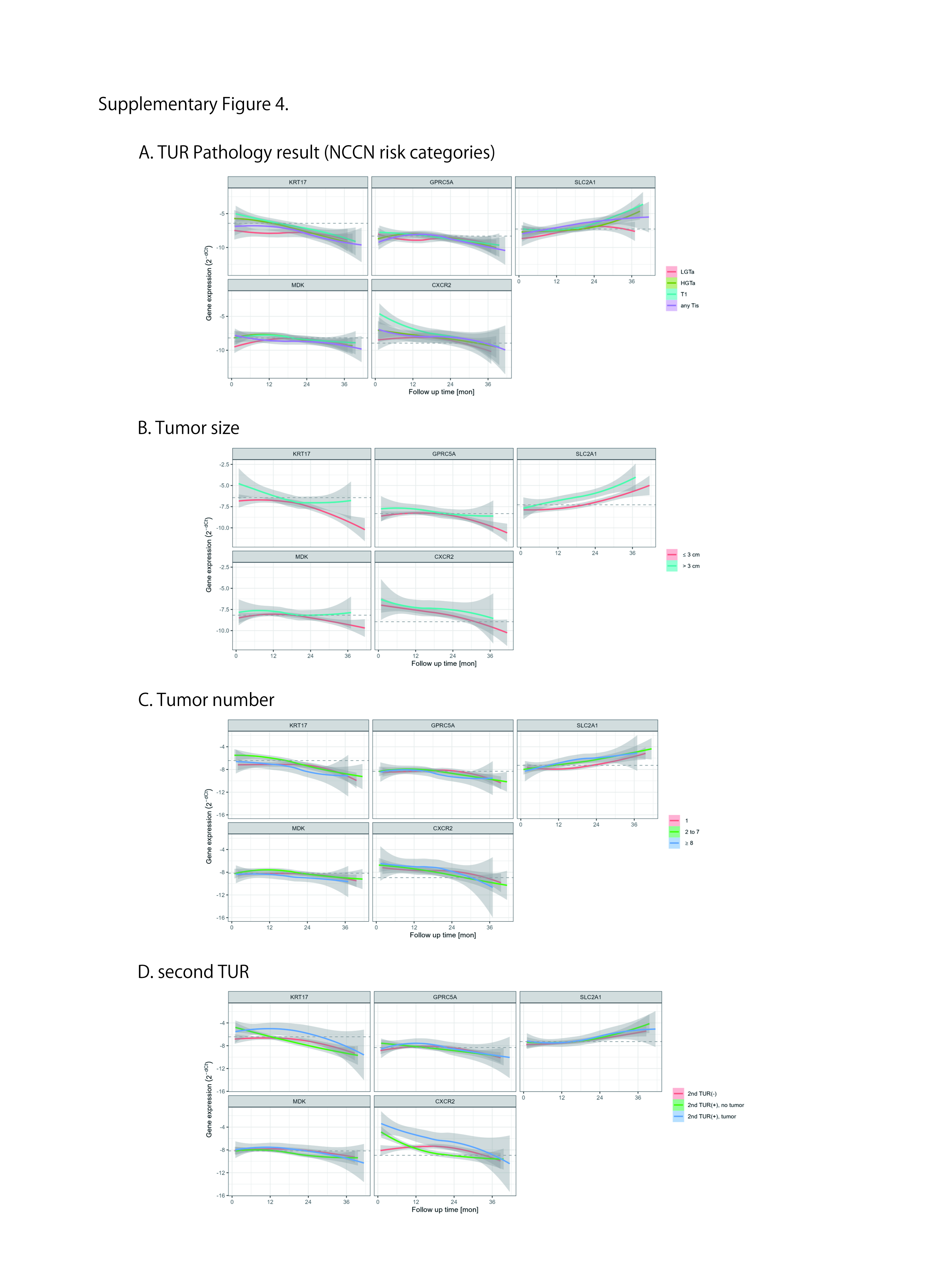

Supplement: Supplementary file 9 — Supplementary Figure 4. [file 41598_2024_55251_MOESM9_ESM.tif]

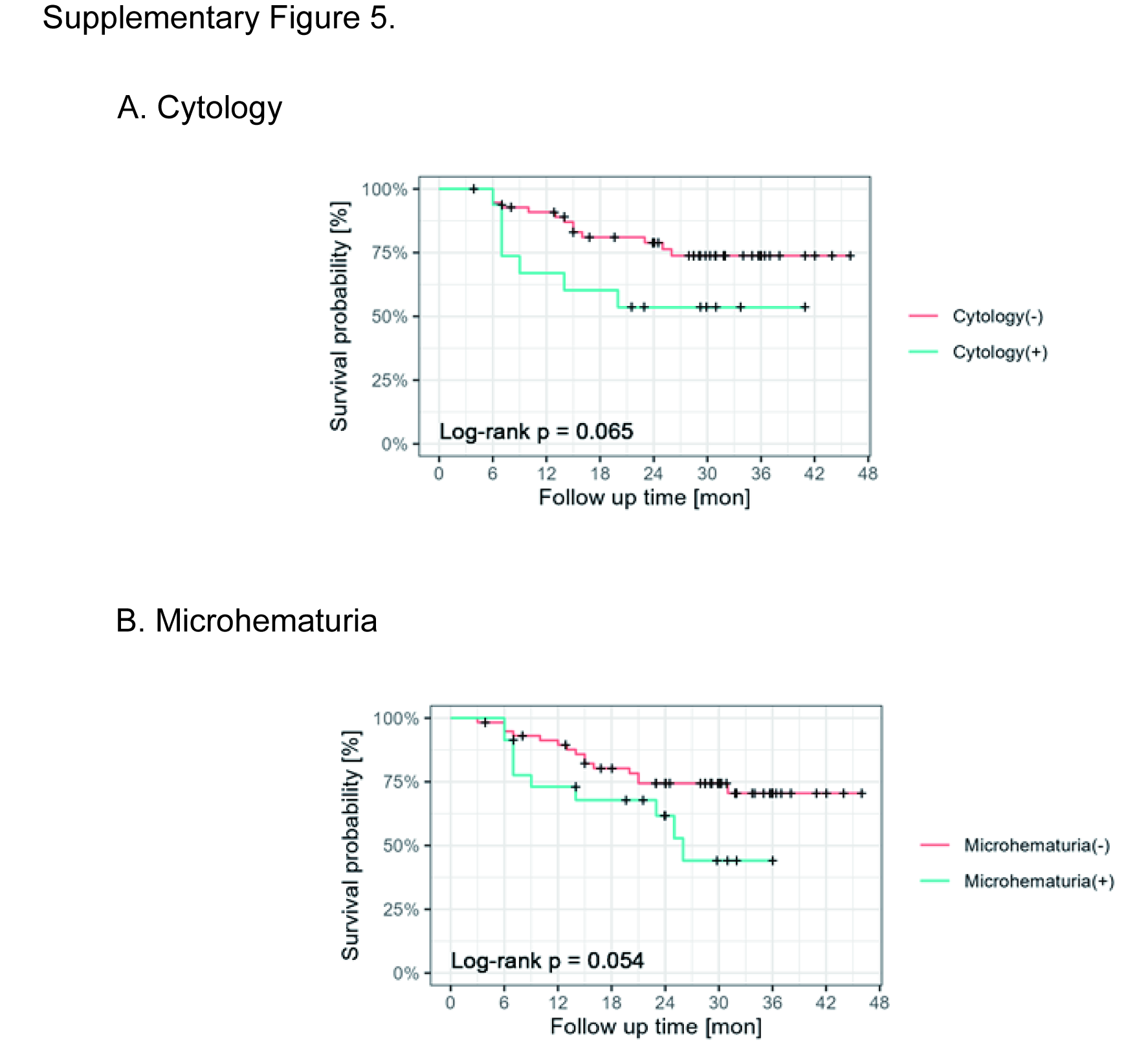

Supplement: Supplementary file 10 — Supplementary Figure 5. [file 41598_2024_55251_MOESM10_ESM.tif]
